# Observing children's outdoor loose parts play, fundamental movement skills, and physical activity in after-school programs through a behavioral mapping approach

**DOI:** 10.3934/publichealth.2026027

**Published:** 2026-04-20

**Authors:** Nila Joshi, Son Truong, Janet Loebach, Daniel Rainham, Becky Feicht, Michelle Stone

**Affiliations:** 1 School of Health and Human Performance, Dalhousie University, Nova Scotia, Canada; 2 Cornell College of Human Ecology, Cornell University, New York, United States of America; 3 Centre for Planetary Health and Sustainable Care, Nova Scotia, Canada

**Keywords:** fundamental movement skills, physical activity, loose parts, outdoor play, behavioral mapping, play spaces, affordances

## Abstract

**Background:**

Outdoor Loose Parts Play (OLPP) has been recognized for its potential to support children's fundamental movement skills (FMS) and physical activity (PA). However, few researchers have explored its association in after-school settings or observed how different outdoor spaces may influence these outcomes.

**Purpose:**

We observed patterns of children's FMS and PA across two after-school programs with varied outdoor spaces in Nova Scotia, Canada, following an OLPP intervention. We also explored how types of loose parts and fixed features were associated with specific FMS and PA intensities.

**Methods:**

Using a multi-site case study approach, behavioral mapping was conducted with children aged 4–12 years pre/post a six-week OLPP intervention. Movements (FMS) were recorded using the Movement Observations during Play (MOP) measure, and PA intensities were recorded using the Children's Activity Rating Scale (CARS). Proportions of FMS, PA intensities, and environmental interactions (LP and fixed features) pre-post were explored using descriptive analyses and McNemar's test (p < 0.05). Logistic regression examined how interaction with LP and fixed features was associated with the likelihood of observing different movements and PA intensities.

**Results:**

Pre-post patterns differed by site. At Site A (larger, suburban, natural outdoor space, pre-existing OLPP), a higher proportion of stability skills were observed, while PA intensity remained unchanged. At Site B (smaller, urban, manufactured outdoor space, limited prior OLPP), a higher proportion of object control skills and moderate-intensity PA were observed. Across both sites, loose manufactured and loose natural materials were more likely to include object control skills, while fixed natural features were more likely to include stability skills.

**Conclusion:**

OLPP was associated with site-specific patterns. Findings suggest that LP types and fixed feature affordances may shape opportunities for different movements and PA intensities. These findings can inform educators and policymakers in designing outdoor play spaces that optimize children's movement opportunities.

## 1. Introduction

Fundamental movement skills (FMS) serve as the cornerstone of children's physical development, enabling them to engage in a wide range of activities with confidence and competence [1]. These skills, which include movements such as running, jumping, throwing, and balancing, are crucial for children's overall development, influencing not only their physical health [2] but also their ability to participate in a wide range of activities such as outdoor play [3]. As children gain proficiency in these skills, they are more likely to participate in regular physical activity (PA), which is essential for promoting long-term health and well-being [4]. The relationship between FMS and PA is intertwined, with one supporting the other in a cycle that fosters physical literacy: *“...the motivation, confidence, physical competence, knowledge, and understanding to value and take responsibility for engagement in physical activities for life”* [5]. Encouraging these behaviors during childhood is particularly important because they set the stage for a lifetime of active engagement and sustained PA participation [6]. One effective way to support FMS and PA is through outdoor play. Outdoor play is inherently engaging [7], particularly when children have access to loose parts (open-ended materials such as wood, tires, balls, buckets, and rope) and offers children the opportunity to practice a wide range of movements in dynamic, real-world contexts. In this paper, we will observe and describe children's FMS and PA in before and after school settings during an outdoor loose parts play (OLPP) intervention using behavioral mapping (BM) as an observational approach. In the following sections, we will explore the relationship between FMS and PA in the context of outdoor play, highlighting how outdoor play can support the development of FMS and PA in children.

### 1.1. Fundamental movement skills: The building blocks of physical activity

FMS are defined as the building blocks of more advanced, complex movements necessary for participation in sports, games, or specific physical activities, and include three key categories: Object control/ball/manipulative skills, locomotor skills, and balance/stability skills [1]. Locomotor skills involve moving the body through space (e.g. walking, running, and jumping); object control skills involve controlling or manipulating objects (e.g. throwing, catching, and kicking); and stability skills involve maintaining balance or posture (e.g. balancing and squatting) [1]. FMS develop through maturation, practice, instruction, habitual activities, such as walking, and physical activities like outdoor play and sports. Through the development of FMS, children can engage in diverse physical activities, enabling them to lead a more active lifestyle [1].

### 1.2. Outdoor play and outdoor loose parts play

Outdoor play provides natural opportunities for children to practice and refine their FMS in a dynamic and engaging environment [8]. One form of outdoor play, loose parts play, involves open-ended materials that can be moved, manipulated, carried, or combined in countless ways, enabling children to explore and create freely [9],[10]. Building on Nicholson's “theory of loose parts” [11], incorporating these materials into outdoor settings can expand affordances available for children's play; this approach is commonly described as OLPP [9]. For this study, the term OLPP will be used to refer to outdoor play with loose parts.

### 1.3. Developmental benefits of outdoor loose parts play

OLPP has been associated with a range of developmental benefits, including cognitive and socio-emotional benefits, by providing children with open-ended materials that support creativity, problem solving, autonomy, and collaborative play [9],[12],[13]. In addition to its cognitive and social-emotional benefits, OLPP also promotes physical development. Environments with natural loose parts have been linked to higher levels of moderate-to-vigorous physical activity [14]. Growing evidence highlights the value of OLPP in supporting children's PA and FMS. The Physical Literacy in the Early Years (PLEY) project (2016–2018) integrated loose parts into outdoor spaces at regulated childcare centers across Nova Scotia to evaluate its impact on physical literacy and active outdoor play. Branje et al. found that OLPP helps children develop FMS, with educators observing increased movement complexity as children engage with these materials [15]. This play encourages exploration, enabling children to practice and refine their movements. Caldwell et al. further emphasized this, noting that repetition of FMS during OLPP enhances physical literacy [16]. While these PLEY findings highlight OLPP's value to FMS development and provide insight into the types of FMS children use while engaging in OLPP, they are based on educators' perceptions and do not provide empirical evidence on how these materials support FMS and PA. Direct observations of children's OLPP could identify the types of FMS children use while engaging with loose parts in outdoor environments, providing a deeper understanding of how the diversity and open-ended nature of loose parts promote different movements.

### 1.4. Outdoor loose parts play in diverse outdoor environments

How children play outdoors in different environments can vary significantly due to the distinct characteristics and resources available in each setting [17]. Given this, we can expect that the way children engage with loose parts can also vary, depending on the design of the outdoor play space, the programming of a center where children are playing, and the environmental features available that enable diverse interactions with the materials. For example, Wishart and colleagues compared children's play behaviors in two outdoor spaces: A more natural environment with loose parts and varied natural features, and a more traditional space with fixed play structures [18]. They found that children in the natural play space experienced a broader range of affordances, utilizing a variety of natural elements for movement-based activities [18]. This implies that it is important to understand the context of each center, such as its environmental features, to assess how these elements support different play behaviors, and the development of FMS and PA.

Many researchers have explored OLPP in early childhood or preschool settings [9],[13],[15],[16],[19],[20], the school setting [21]–[24], and park settings [25],[26]. However, our knowledge, there is no research on OLPP in after-school settings, and more specifically, how OLPP in after-school settings contributes to children's FMS and PA. This is important considering the significant amount of time children spend in after-school programs [27], which provide an additional opportunity for them to develop FMS and engage in PA. Given the limited research on OLPP in after-school settings and the lack of studies comparing how children's FMS and PA during OLPP vary across outdoor environments, it is important to understand how environmental features shape children's interactions with these materials. The way play spaces are structured may offer unique opportunities for engagement. This brings us to the concept of affordance theory, which helps explain how features of the environment can support children's PA and FMS development when engaging with loose parts.

### 1.5. Affordance theory and outdoor loose parts play

Affordance theory, originally developed by psychologist James J. Gibson, offers a framework for understanding how children perceive and interact with their environment and the outdoor play space [28]. According to Gibson, affordances are the opportunities for action that the environment provides, which are directly perceived by individuals based on their capabilities and needs. For example, a tree may afford climbing for a child, while a flat surface may afford walking. In the context of outdoor play, affordance theory provides a lens through which to understand how the physical environment shapes children's play behaviors and supports aspects of their development [29]. Heft's taxonomy of environmental affordances [30] and Kyttä's work on mobility highlight how children's interactions with outdoor settings are shaped by these contextual features and their developmental needs [31]. To better understand how OLPP promotes FMS and PA in real-world settings, observational methods are essential.

### 1.6. Behavioral mapping

We can better understand children's outdoor play behaviors and their interactions with their environments through systematic observation. One approach to systematic observation is behavioral mapping (BM), which involves direct observation and recording of behaviors occurring in a particular setting at a specific time [32]. In the context of outdoor play, BM can be used to observe and record play behaviors within the outdoor play space and identify features that facilitate the child's play, along with any social or individual factors being exhibited [18],[25],[32]. BM offers a more detailed approach by documenting children's behaviors in real-time, providing a clearer understanding of how interventions, such as the introduction of loose parts, can influence FMS and PA across environments. While BM has been used to study outdoor play, a significant gap remains in understanding how OLPP influences children's PA and FMS, particularly within the after-school setting. Additionally, more experimental research is needed to explore and describe the association between OLPP and children's PA and FMS, providing quantitative evidence on how these materials contribute to children's physical development.

### 1.7. Study purpose

Our purpose of this study was to observe and describe how the introduction of an OLPP intervention, situated within two after-school programs in Nova Scotia, Canada, was associated with children's observed movements (use of FMS: locomotor, object control, and stability skills) and PA intensity. Using BM to record children's outdoor play events before and after the integration of loose parts into the outdoor play environments, we were interested in exploring whether the proportion of observations in which children used different movements [FMS: (locomotor, object control, and stability skills)] and PA intensity levels (ranging from stationary to fast movement) within play events differed across time points and how the types of loose parts (loose manufactured, loose natural) and fixed features (fixed manufactured, fixed natural) were associated with the likelihood of observing specific FMS and PA intensity levels.

## 2. Methods

### 2.1. Study design

This study is part of a larger research project, the Physical Literacy in the Early Years (PLEY) School project. The purpose of the PLEY School project was to examine the impact of a 6-week OLPP intervention on children's physical literacy in three before- and after-school programs within the Halifax Regional Municipality in Nova Scotia, Canada. A case study approach was chosen to provide an in-depth analysis of how the intervention influenced children's physical literacy development at each site. Before- and after-school programs were selected to ensure diversity in location (urban or suburban) and socioeconomic status. To be eligible, programs needed to include children from pre-primary to grade 6 (age 4 to 12 years) in their before-and-after school programming.

Loose parts were integrated into the outdoor spaces of the programs (specifically, the after-school programs) in the summer of 2022; observations of children's play were conducted at baseline (Pre-intervention, T0, May 2022) and post-intervention (Weeks 5 and 6, T6, June to July 2022). A description of the intervention, including outcomes of interest and measures, is provided below. The project was approved by Dalhousie University's Research Ethics Board (REB #2021–5783). In this paper, we examined data from two of the three before-and-after-school programs included in the broader PLEY School project, using a multi-site case study approach, analyzing data from each site independently to explore and describe patterns associated with the intervention (given the distinct characteristics of each site). While cross-site comparisons highlighted differences and similarities, direct comparisons between the two sites were not conducted due to the distinct contextual and environmental differences, which made a direct, one-to-one comparison not suitable.

### 2.2. Participants

Participants included children aged 4 to 12 years attending after-school programming at the designated sites. Informed written consent was obtained from parents/legal guardians of all participating children. At Site A, 43 children (25 boys and 18 girls) were recruited at T0, with 38 (22 boys and 16 girls) participating in T6. At Site B, 16 children (10 boys and 6 girls) were recruited at T0 and 9 (6 boys and 3 girls) participated in T6. A significant number of dropouts occurred as data collection transitioned to the summer months. Many children who initially participated at baseline were unable to continue due to not attending the after-school program during that period.

### 2.3. Outdoor loose parts play intervention

**Figure 1.**
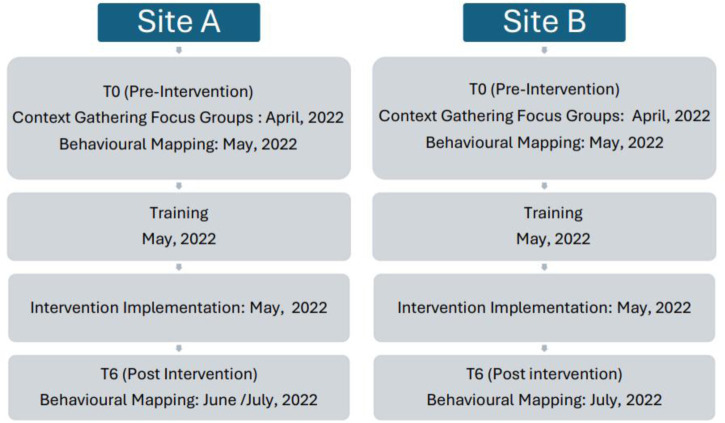
Timeline of data collection and intervention start for Site A and Site B.

**Figure 2.**
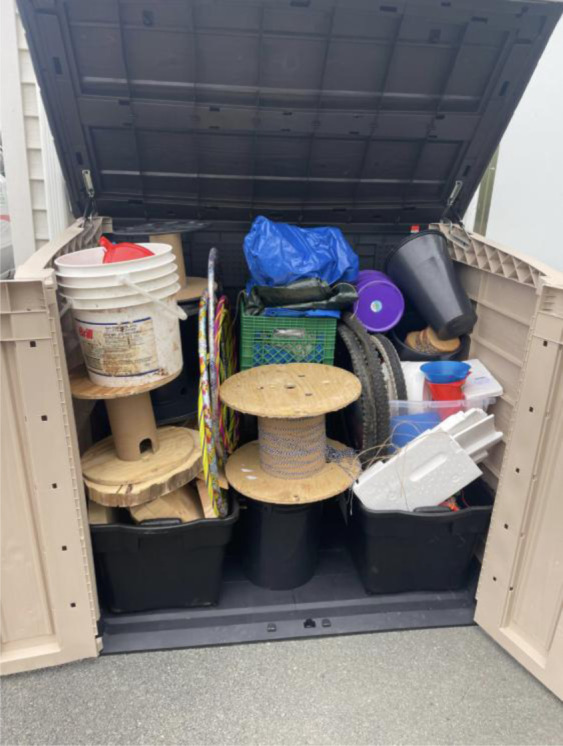
Example of a loose parts kit provided to participating sites.

The PLEY School project intervention components and associated timeline are provided below. Context-gathering focus groups with program staff and the site director were conducted on April 5, 2022, for Site A and on April 11, 2022, for Site B. These sessions gathered information about each site's programming, their practices related to outdoor play, the culture of play within their center, and details about their outdoor play space. Photos were also taken of the sites' outdoor play spaces. Following these sessions, baseline BM data collection commenced (May 2022) to gather information on how children played outdoors before the implementation of the OLPP intervention (described below). Educational training on key elements of the project was then provided to program staff via virtual webinars through Microsoft Teams (May 2022); topics included OLPP, active outdoor play, physical literacy, FMS, and the larger PLEY School project. After each site completed all six webinars, loose parts were introduced into their outdoor space, marking the beginning of the 6-week intervention (see Figure 1: Timeline; Site A – loose parts delivered on May 17th, 2022; Site B – loose parts delivered on May 30th, 2022). Each site received the same loose parts kit. The items included were determined through consultation with directors from the before-and-after school sites and through a review of existing loose parts literature. Through these consultations, sites identified the types of materials children at their centers currently engage with, as well as those they do not, but may potentially, use. Through the review of the literature, the emphasis on diversity in natural and manufactured loose parts was highlighted, particularly in terms of their potential to engage children in varied play experiences [10],[33]. As such, the loose parts kit consisted of natural and man-made materials (tree cookies, pool noodles, tarps, logs and long wood, balls, piping and tubing, wooden planks, rope, buckets, hula hoops, and tires). An example of the loose parts kits provided to sites is shown in Figure 2.

### 2.4. Study sites

The study was conducted in two licensed after-school programs in Nova Scotia, Canada, selected to represent contrasting outdoor play environments: a suburban, nature-rich site (Site A) and an urban, predominantly built outdoor site (Site B). Photographs included in the manuscript depict the outdoor play areas used for BM at each site and were taken by the research team during site visits conducted as part of recruitment, with identifying details removed to protect site anonymity.

#### 2.4.1. Site A

*After-School Program Description*: This center includes pre-primary to grade 5 students (ages 4 to 11). The program is comprised of mixed-aged groups (n = 4); each group consists of at least 20 children and is supervised by 3–4 educators. All staff members are required to have completed the Before and After Pre-Primary (BAPP) training, which focuses on supporting children's movement and physical literacy through outdoor play [34]. Approximately 90% of the after-school programming (90 minutes) is allocated to outdoor play, with at least two-thirds of that time (60 minutes) devoted to engaging children in PA.

**Figure 3.**
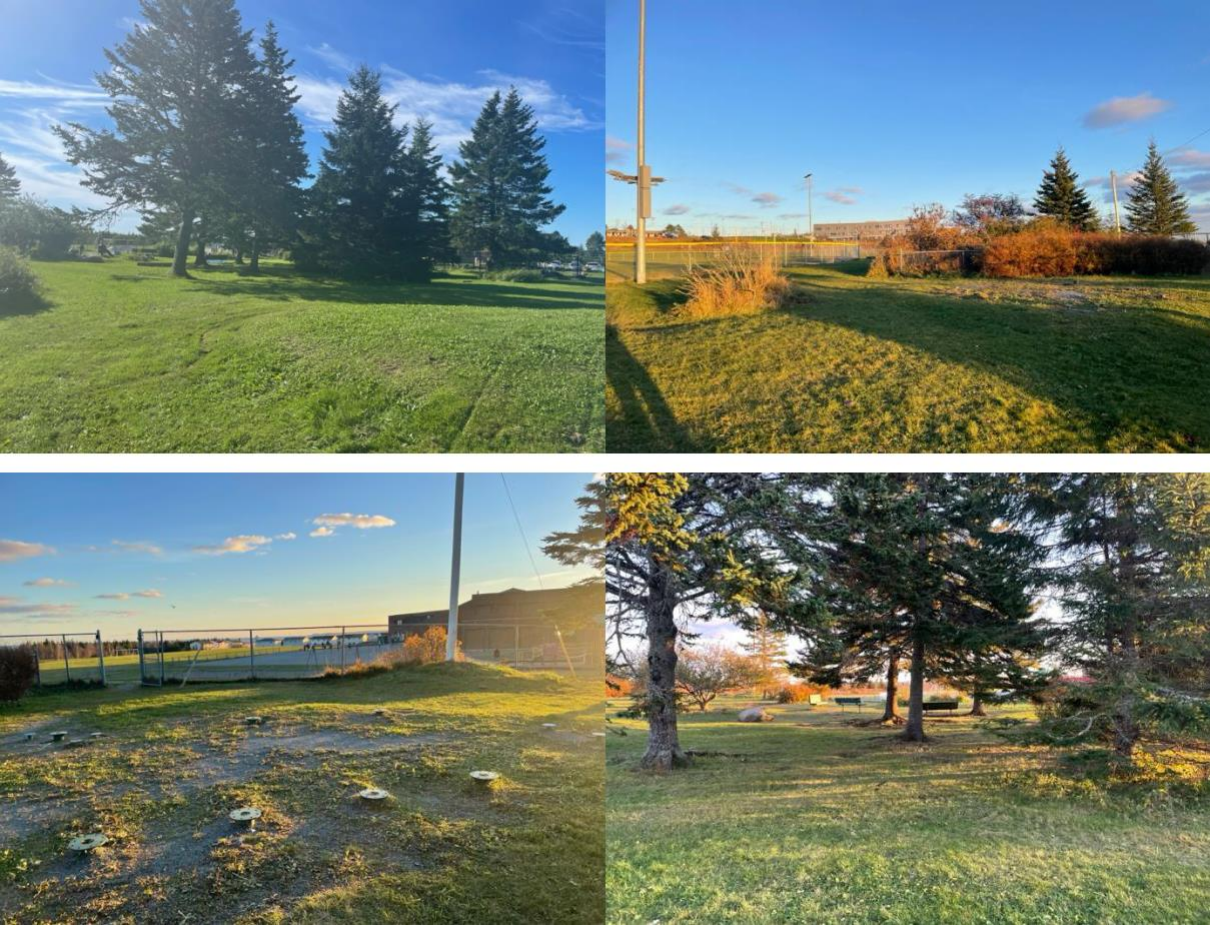
Site A Outdoor Space Features. Clockwise from Top Left: Photo 1: Open green space with trees; Photo 2: Open green space with bushes; Photo 3: Gravel and sandpit; Photo 4: Treed area.

*Outdoor Play Space Description*: Initial meetings with the site during the recruitment period and context-gathering focus groups provided an opportunity to gain a deeper understanding of the outdoor play space and typical play activities children engage in. The site features several key areas, including a fixed playground, a large green space, a soccer field, a baseball field, and proximity to the ocean. The children engage in diverse outdoor experiences, exploring the woods, participating in gardening activities, and utilizing loose parts. Additionally, the site includes an unfenced garden, a large open area with trees, bushes, and rocks, primarily used by the school-aged children. The after-school program often brings loose parts to this play space. These materials are diverse and include sports equipment (e.g. balls, frisbees), kitchen utensils (e.g. spoons, bowls), and natural loose parts (e.g. wood, sticks). The center has a large storage space dedicated to storing loose parts. The forest area spans approximately 2000–3000 square feet. Example photographs of the Site A play space are presented in Figure 3.

#### 2.4.2. Site B

*After-School Program Description*: This site is an after-school program that offers more structured programming as part of its partnership with a community-based organization. Moreover, this organization is dedicated to providing a safe and supportive environment for children and youth. Unlike the previous site, this program follows a more structured approach to its scheduling, with specific activities assigned to each day of the week (e.g., Art on Mondays, Science, Technology, Engineering, and Math (STEM) on Tuesdays). The children are divided into three age groups: 5–6 years old, 7–8 years old, and 9–12 years old. Each age group is given specific intervals for outdoor play, in accordance with the daily schedule.

**Figure 4.**
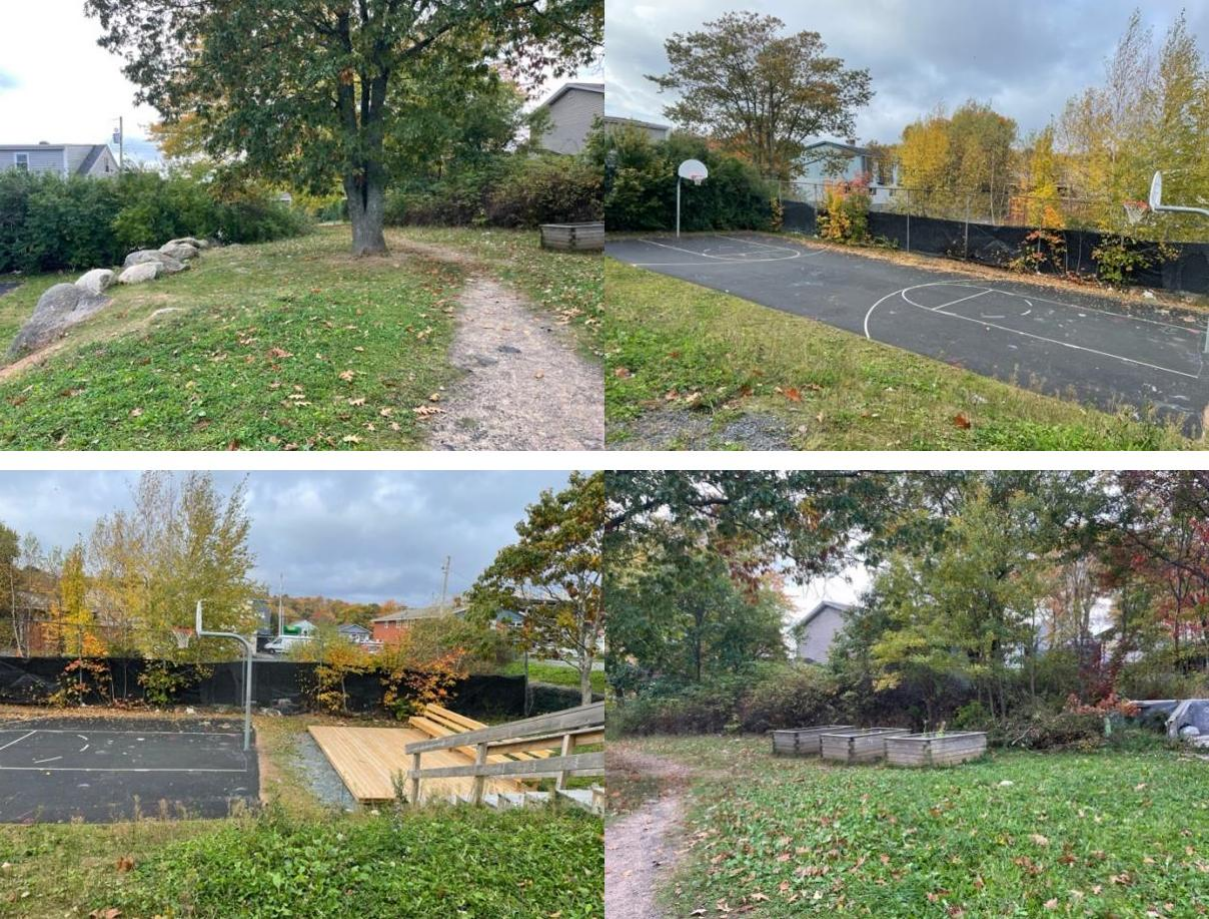
Site B Outdoor Space Features. Clockwise from Top Left: Photo 1: Tree and walking path; Photo 2: Open asphalt area (basketball court); Photo 3: Elevated wooden platform and bench; and Photo 4: Three wooden garden boxes.

*Outdoor Play Space Description*: The site includes a renovated (2021) basketball court where the children actively participate in basketball, soccer, and unstructured free play activities (e.g. running and tag). Alongside the basketball court, there is a large wooden platform containing a bench seating area. Additionally, there is a large parking lot that serves as a play area, and a small section of green space with garden boxes where the children also engage in play. Prior to participating in this study, the site's use of loose parts in the outdoor play space was quite limited, consisting primarily of manufactured materials (e.g. basketballs and soccer balls). These materials were mainly used for structured activities rather than being integrated into unstructured loose parts play, which was not commonly observed at the site. Example photographs of the Site B play space are presented in Figure 4.

### 2.5. Data collection

#### 2.5.1. Behavioral mapping protocol

A place-based BM approach was used to observe outdoor play events. This method helps to understand children's PA and FMS by focusing on the geographical setting where these behaviors occur [35]. Unlike a person-based approach, which concentrates on the activities of a single individual, place-based BM captures how the play space is utilized [35]. This approach provides insights into the dynamics of the setting, rather than tracking the behavior of a specific person over time. By using place-based BM, we can gain a better understanding of how the outdoor play space with loose parts influences children's PA and FMS.

#### 2.5.2. Data collection preparation

A training session was conducted with the field team, which included two PhD students (JC1, NJ), an undergraduate research assistant (KM), and a retired early childhood educator (JC2). Led by research team members (JL, AC), these sessions focused on BM, including its methodology and application to specific research questions. Based on this training and a review of existing tools, the decision was made to design a new observational measure to capture diverse FMS during outdoor play, specifically, OLPP, resulting in the Movement Observations during Play (MOP) tool [36]. In addition to FMS, the Children's Activity Rating Scale (CARS) was selected to evaluate the intensity of PA observed during these outdoor play events [37]. In consultation with members of the research team (JL, AC), the decision was made to conduct a total of 10 hours of observations at each time point (T0, T6), for each site, using a 15-second scan protocol.

Once the measures were finalized, the field team completed approximately 25 hours of video-based training to practice coding FMS, PA, and environmental interactions (use of loose parts). This was followed by 12 hours of reliability rounds at the participating sites to ensure consistency and accuracy [36]. During reliability rounds, two observers independently coded the same play-event scans (15-s intervals) within the same observation sessions, and agreement was assessed for key variables (MOP Movements, CARS intensity categories, and loose parts/fixed-feature codes). Inter-rater reliability was assessed using Cohen's kappa. The MOP measure enables recording up to five movements within a single 15-second scan; however, during piloting, the raters most consistently identified one to two movements per scan (κ = 0.78–0.96). Acceptable agreement was observed for CARS (κ = 0.70) and high agreement for loose parts coding (κ = 0.87). Observations were recorded using the ArcGIS Field Maps application [38] on a Samsung tablet.

#### 2.5.3. Observation areas

**Figure 5.**
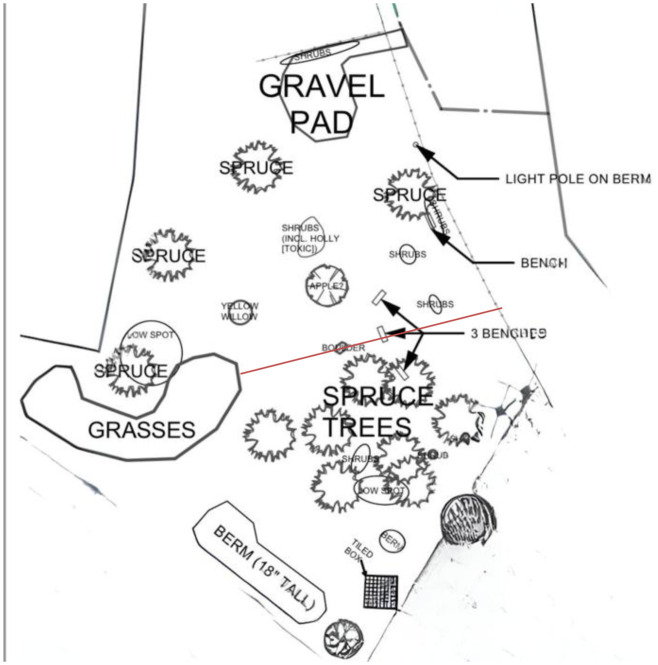
Basemap of Site A. Note: The map depicts the primary natural features (e.g., spruce trees and grasses), built elements (e.g., benches), and the boundary used to divide the space into two observation zones for BM. The red line indicates the zone boundary. This basemap was used to geolocate play-events observed.

**Figure 6.**
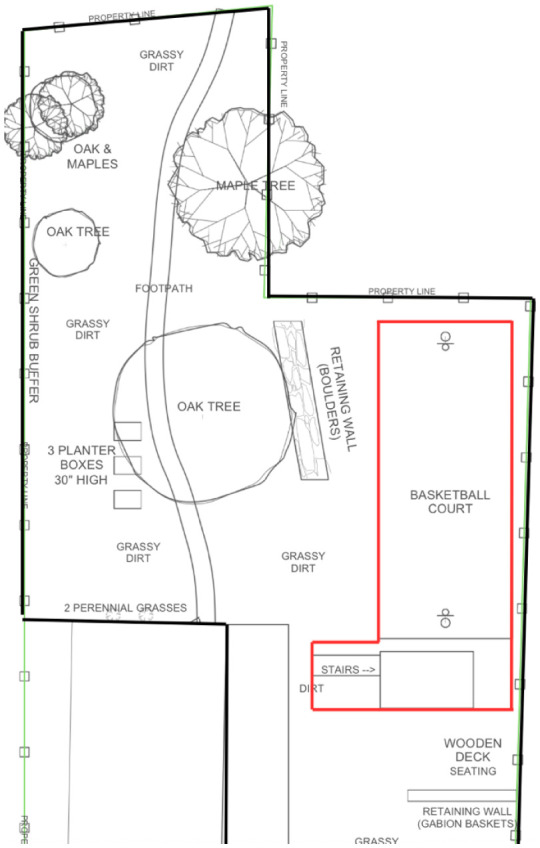
Basemap of Site B. Note: The map illustrates a predominantly manufactured play environment, including the basketball court and adjacent wooden platform and bench, alongside the surrounding green space and perimeter areas. The red outline indicates the boundary used to divide the site into two observation zones for behavioral mapping (Zone 1: basketball court/wooden deck and bench; Zone 2: green space/perimeter). This basemap was used to geolocate play-events observed.

Observations were conducted in two designated zones at each site, which were identified through site visits and consultations with directors regarding the areas where children played the most. At Site A, a large green space with various natural features such as trees and bushes, the observation area was divided by a prominent boulder, which served as a natural marker for the midpoint of the play space (Figure 5). At Site B, the observation zones were defined by the primary play areas: The basketball court (including the adjacent wooden deck with a bench) and the surrounding green space and parking lot (Figure 6).

#### 2.5.4. Recording procedures

Several members of the field team recorded observations of children's outdoor play (i.e., play event) at the two sites (NJ, JC1, JC2, KM). Data were collected over two weeks at each time point (T0, T6). Observations were scheduled during the after-school program between 3:00 and 5:30 PM. Each time point included five days of observations, with each day consisting of 2 hours of data collection, resulting in a total of 10 observer hours. Pre-intervention (T0) observations took place in May 2022, while post-intervention (T6) observations occurred in June and July 2022. Observations were conducted only during good weather conditions, either cloudy or sunny, ranging from 15–20 °C in May and 20–30 °C in June and July, and never on rainy days to protect the tablets used for data collection.

#### 2.5.5. Data attributes collected

During pre-intervention data collection (T0), colored bands were distributed to children whose parents had consented to their participation in the study. As observers became familiar with participants, bands were no longer used in the final data collection (T6). Grades were grouped as: Pre-Primary/Primary, Grades 1–3, and Grade 4–6.

FMS were coded using the MOP observational measure, a typology and protocol which was specifically developed to better capture and understand children's FMS during outdoor play and OLPP [36] (see Appendix). The observational measure included 46 movements, organized into three FMS categories: locomotor (n = 19), object control (n = 15), and stability (n = 12). Up to five movements could be recorded per play observation. PA intensity was observed and coded using the CARS, which assessed energy expenditure for young children based on observed behaviors [37]. The scale included five categories from 1 (stationary-motionless) up to 5 (fast movement). Environmental interaction data included observations about the involvement of fixed features or loose parts as part of the play event. Up to four environmental interactions could be coded for each observation and were based on whether the item was fixed (immovable) or loose (movable), and whether or not the item was in its natural form or manufactured. Specific loose parts and fixed features that were being used in observational scans were written down in an open text box.

#### 2.5.6. Observation procedure

At Site A, participating children played in designated areas previously used for loose parts play, while non-participating children played elsewhere. This arrangement was organized by the site director and program staff. At Site B, all children played together, regardless of their participation in the study.

Observations took place during regular after-school outdoor time (after 3:00 PM), depending on each site's schedule. Two observers collected data at T0 (pre-intervention) and T6 (post-intervention), each responsible for one of two distinct zones per site, identified during prior site visits. Observations were recorded on tablets using the ArcGIS software application “Field Maps” [38], a geographic information system commonly utilized in BM research. MOP [36] and the CARS tool [37] were uploaded into the Field Maps application, enabling observers to accurately record the locations of play activities. Observations were completed only on children with consent.

The observation process began with the researcher scanning the designated zone in a clockwise direction until a child came into view. The child's FMS and PA were observed for 15 seconds, followed by up to 1 minute of recording observations onto the tablet. The observer then resumed scanning from the last observation point. If no new children appeared, the scan continued, and any visible children were coded. This iterative process ensured thorough observation of all children in the zone. Observers switched after the first hour to prevent habituation and take a break.

### 2.6. Data analysis

All data were downloaded to an ArcGIS account and loaded into ArcGIS Pro Version 2 [38]. Observational data were then exported from ArcGIS into Microsoft Excel and SPSS for further quantitative analysis. All statistical analyses were conducted using SPSS Version 28 [39]. A descriptive analysis was used to examine the proportions of FMS, PA intensities, and environmental interactions (fixed features and loose parts) pre- and post-intervention (T0, T6). Loose parts use was summarized by loose part type (loose manufactured vs. loose natural). Because children could interact with both types within the same play observation, counts for loose manufactured and loose natural materials were tallied separately; therefore, when combined, the total may exceed 100% because both types could be observed in the same play event. McNemar's test was used to determine whether the changes in proportions were significant. Binary logistic regression was used, and odds ratios were calculated, to assess how children's engagement with loose parts and fixed features was associated with the likelihood of them engaging in different PA intensities (stationary-fast movement) and different FMS (locomotor, object control, and stability skills).

The unit of analysis was the observational scan (play event) recorded during BM. Scans were collected repeatedly within each site at T0 and T6 and were analyzed at the observation level within the site. Because children were not individually identified, scans could not be linked to specific children; therefore, repeated measures within children could not be modeled, and inferential analyses treated scans as independent observations within each site. As a result, odds ratios were interpreted as observation-level associations rather than child-level effects. For the same reason, within-child clustering could not be assessed or adjusted for (e.g., using clustered standard errors, generalized estimating equations, or multilevel models).

The data were organized in SPSS, matching all observed FMS and PA intensities with the types of loose parts and fixed features present during each observational scan. The loose parts were categorized as loose manufactured or loose natural, and fixed features as fixed manufactured and fixed natural. For the analysis, if a loose part or fixed feature was present during the scan, it was coded as 1; if it was absent, it was coded as 0. When calculating the odds ratios, the FMS or PA intensity of interest, such as locomotor skills or slow, easy movements, was assigned a value of 1, while all other FMS or PA intensities were coded as 0. For example, when analyzing locomotor skills, all observed locomotor skills were coded as 1, and all other FMS (object control and stability) were coded as 0. In this analysis, locomotor skills were entered as the dependent variable, and the categories of loose parts and fixed features were included as covariates. The logistic regression model was then executed, and the resulting odds ratios were reported. Statistical significance was determined at a p-value of <0.05.

## 3. Results

### 3.1. Play events

At Site A, a total of 458 play events (observations) were captured pre-intervention (prior to the integration of loose parts; T0), with 612 play events captured post-intervention (post integration of loose parts; T6). In comparison, 660 play events were captured pre-intervention at Site B, with 563 recorded post-intervention. At both sites and both time points, boys were captured in a significantly greater proportion of observations (Site A: T0 = 54.6%, T6 = 55.1%, (p < 0.001); Site B: T0 = 73.0%, T6 = 67.5%, (p < 0.001) (Table 1). Similarly, in both time points, participants in Grade 1–Grade 3 were captured in a significantly greater proportion of observations (Site A: T0 = 63.9%, T6 = 83.8%, (p < 0.001); Site B: T0 = 88.6%, T6 = 70.9%, (p < 0.001). At Site A, the proportion of play events involving boys and girls did not change significantly pre- to post-integration of loose parts, but did at Site B, with boys being represented in slightly fewer play events at T6 than T0 (67.5% vs. 73.0%). From T0 to T6, the proportion of play events involving Grade 1–3's increased at Site A (from 63.9% to 83.8%) yet decreased at Site B (from 88.6% to 70.9%), with fewer Pre-Primary-Primary and Grade 4–6 at T6 vs. T0 (Site A) and more Pre-Primary to Primary at T6 vs. T0 (Site B) (p < 0.001).

### 3.2. Changes in observed fundamental movement skills

Observations of FMS using the MOP also revealed variation in the types of FMS children were using, within and between sites, and over time (Table 1). Before the introduction of loose parts at Site A, object control skills were observed in 71.6% of play events, stability skills in 62.7%, and locomotor skills in 54.2%. Once loose parts were introduced to the outdoor play space, the proportion of play events involving object control skills decreased (to 64.4%, p < 0.001). In comparison, the proportion of play events involving stability skills increased (to 65.5%, p = 0.002). At Site B, just over 70% of play events at T0 (pre-loose parts integration) included locomotor skills, with 60.6% and 44.6% including stability and object control skills, respectively. Once loose parts were introduced into the outdoor play space, the proportion of play events involving locomotor skills and stability skills decreased (to 69.4% and 55.6%, respectively, p < 0.001); the proportion of play events involving object control skills significantly increased (to 74.9%, p < 0.001).

### 3.3. Changes in observed physical activity intensity

Observations of PA intensity using the CARS revealed some variation in the intensity of play events, within and between sites, and over time (pre- to post-intervention) (Table 1). At both sites and both time points (T0, T6), most play events were of light and moderate intensity (stationary with limb movement, slow/easy, moderate); less than 3% of play events were classified as stationary-motionless activity and less than 10% were classified as fast movement. At Site A, the intensity of play events did not change significantly once loose parts were introduced into the outdoor play space; proportions of play events in each PA intensity category were similar at T0 and T6. At Site B, following the introduction of loose parts, there was a slight reduction in the number of play events classified as stationary motionless activity (T0 = 2.7%, T6 = 0.8%, p = 0.04) and a more prominent reduction in the number classified as slow, easy movement (T0 = 46.1%, T6 = 28.6%, p < 0.001), with the proportion of moderate intensity play events increasing significantly (T0 = 22.3%, T6 = 46.0%, p < 0.001).

### 3.4. Variation in observed loose parts use

Observations of loose parts used within play events also revealed variations in the categories and specific types of loose parts children were using within and between sites and over time (Table 1). At Site A, before the intervention (T0), just over two-thirds of play events involved loose manufactured materials; of these, noodles (17.8%), pipes (15.8%), and bowls (11.5%) were the most common items used. Just over 20% of play events included the use of natural loose parts, with 23.7% of those involving the use of dirt. A total of 22.2% of play events involved fixed natural features; 90.2% of those involved an interaction with trees. A total of 3.9% of play events involved fixed manufactured features, with half of these involving benches. Following the intervention, the proportion of play events involving loose manufactured items decreased slightly (to 55.4%), yet the change pre-post was not significant (p = 0.183); here, planks (18.6%), tarps (9.4%), and string (7.7 %) were the major materials used. The proportion of play events involving loose natural materials decreased (to 18.8%, p = 0.02), with 40% of these play events involving the use of sticks. The proportion of play events involving fixed natural features increased post-intervention (to 29.7%, p = 0.007), again with most (94.5%) involving interactions with trees. Interactions with fixed manufactured features did not change significantly pre-post intervention; again, most (82.8%) involved the use of benches.

**Table 1.** Descriptions for all observed play events pre-intervention (T0) and post-intervention (T6).

	Site A					Site B				
	
	Pre-intervention (T0)		Post-intervention (T6)			Pre-intervention (T0)		Post-intervention (T6)	
	# of play events	% of play events	# of play events	% of play events	P value	# of play events	% of play events	# of play events	% of play events	P value
Total Play Events	458		612			660		563		
Gender										
Male	250	54.6%	337	55.1%	0.653	482	73.0%	380	67.5%	0.03
Female	206	44.9%	274	44.8%	0.796	175	26.5%	181	32.1%	0.026
Null/Incomplete data	2	0.4%	1	0.2%	/	3	0.5%	2	0.4%	/
Grade										
Pre-primary-Primary	74	16.1%	41	6.7%	<0.001	66	10%	144	25.6%	<0.001
Grade 1-Grade 3	293	63.9%	513	83.8%	<0.001	585	88.6%	399	70.9%	<0.001
Grade 4-Grade 6	90	19.7%	51	8.3%	<0.001	0	0	9	1.6%	/
Null/Incomplete data	1	0.2%	7	1.1%	/	9	1.7%	11	2.0%	/
Fundamental Movements Skills (MOP)			
Locomotor skills	249	54.2%	362	59.2%	0.17	463	70.2%	391	69.4%	<0.001
Object control skills	328	71.6%	394	64.4%	<0.001	294	44.6%	422	74.9%	<0.001
Stability skills	287	62.7%	401	65.5%	0.002	400	60.6%	313	55.6%	<0.001
Null/Incomplete data										
Physical Activity Intensity (CARS)			
Stationary (motionless)	13	2.8%	8	1.3%	0.26	18	2.7%	5	0.8%	0.04
Stationary (limb movement)	117	25.5%	118	19.3%	0.15	132	20%	91	16.2%	0.17
Slow easy	182	39.7%	273	44.6%	0.12	304	46.1%	161	28.6%	<0.001
Moderate	105	22.9%	168	27.5%	0.30	147	22.3%	259	46.0%	<0.001
Fast	35	7.6%	38	6.2%	0.17	55	8.3%	41	7.3%	0.91
Null/Incomplete data	6	1.3%	7	1.1%	/	4	0.6%	6	1.1%	/
Loose Parts & Fixed Features			
Loose Natural	114	24.9%	115	18.8%	0.022	66	10%	55	9.8%	0.702
Loose Manufactured	304	66.4%	339	55.4%	0.183	268	40.6%	445	79.0%	<0.001
Fixed Natural	102	22.2%	182	29.7%	0.007	20	3.0%	22	3.9%	0.522
Fixed Manufactured	18	3.9%	29	4.7%	0.743	84	12.7%	69	12.3%	0.661
No loose parts	61	13.3%	80	13.1%	0.261	243	36.8%	63	11.2%	<0.001
Null/incomplete data										

Note: Statistical significance was set at p < 0.05. “/” indicates no data available.

At Site B, prior to the intervention, 40.6% of play events involved the use of manufactured loose parts, with fixed manufactured features, loose natural materials, and fixed natural features being used in 12.7%, 10%, and 3% of play events, respectively. Balls (23.5%), skipping ropes (16.8%), and basketballs (16.8%) were the most common loose manufactured items documented, with benches (47.6%), sticks and flowers (28.8%, 13.6%), and trees (70%) being the most common fixed manufactured features, loose natural materials, and fixed natural features children engaged with. Following the intervention, the proportion of play events involving loose manufactured materials increased (to 79.0%, p ≤ 0.001), with tires (18.2%), pool noodles (12.1%), and basketballs (11.2%) being the major materials used. The proportion of play events involving fixed natural and manufactured features and loose natural materials, did not change significantly pre-post intervention, with trees (77.2%), mud kitchens and garden boxes (69.8%), and mud and flowers (32.7%) being the most common fixed natural and manufactured features and loose natural materials children engaged with, respectively. Finally, there was a significant decrease in the proportion of play events involving no loose parts from T0 (36.8%) to T6 (11.2%) (p < 0.001).

**Table 2.** Associations of loose parts and fixed feature use with fundamental movement skills.

	Site A				Site B		
FMS Category	Type of Loose Part or Fixed Feature	Odds Ratio	Lower Bound	Upper Bound	Odds Ratio	Lower Bound	Upper Bound
Locomotor	Loose Manufactured	0.49*	0.37	0.63	0.28*	0.21	0.38
	Loose Natural	0.40*	0.29	0.55	0.30*	0.20	0.45
	Fixed Manufactured	0.39*	0.21	0.73	0.76	0.55	1.06
	Fixed Natural	0.26*	0.20	0.35	0.61	0.33	1.10
Object Control	Loose Manufactured	3.36*	2.59	4.37	5.50*	3.82	7.93
	Loose Natural	3.09*	2.29	4.16	2.29*	1.62	3.22
	Fixed Manufactured	0.84	0.47	1.49	0.83	0.60	1.14
	Fixed Natural	0.87	0.67	1.12	0.50*	0.27	0.92
Stability	Loose Manufactured	0.58*	0.46	0.74	0.74	0.55	1.00
	Loose Natural	0.73*	0.54	0.99	1.28	0.90	1.80
	Fixed Manufactured	2.48*	1.49	4.14	1.61*	1.17	2.21
	Fixed Natural	3.15*	2.47	4.02	2.84*	1.68	4.78

Note: An odds ratio (OR) of 1 indicates no association. An OR greater than 1 suggests a positive association and higher odds, while an OR less than 1 indicates a negative association with lower odds. * p < 0.05.

### 3.5. Loose parts and movement: likelihood of fundamental movement skills post-intervention

**Figure 7.**
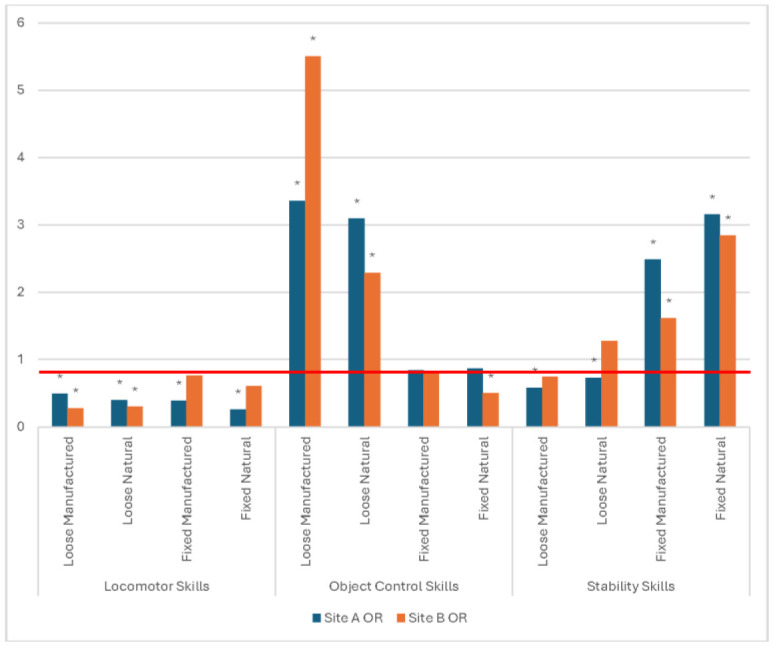
Odds ratios of loose parts and fixed feature use by fundamental movement skills. Note: The y-axis displays odds ratios (unitless). Bars marked with an asterisk (*) indicate statistical significance (p < 0.05). Bars above the red line (OR > 1) represent increased odds, bars below the red line (OR < 1) indicate reduced odds, and bars at the red line (OR = 1) represent no association.

Table 2 and Figure 7 present the odds ratios for the likelihood of specific types of loose parts and fixed features being associated with the three types of FMS (locomotor skills, object control skills, and stability skills) in play observations captured post-intervention (T6). Across both sites, locomotor skills were generally less likely to occur during play events involving loose parts, indicating a shift away from locomotor movements when materials were being actively used. In contrast, object-control skills were more strongly linked to loose parts, particularly when children engaged with loose materials rather than fixed features. Stability skills showed a different pattern: Stability was most consistently associated with fixed features (both manufactured and natural). Notably, loose parts tended to correspond with a lower likelihood of stability skills relative to fixed-feature play, reinforcing that loose-part play may emphasize manipulation and construction-oriented movements more than balance-focused movements. Site-level patterns were broadly similar, with Site B showing especially strong associations between loose manufactured materials and object-control skills.

### 3.6. Loose parts and physical activity: Likelihood of physical activity intensity post-intervention

**Figure 8.**
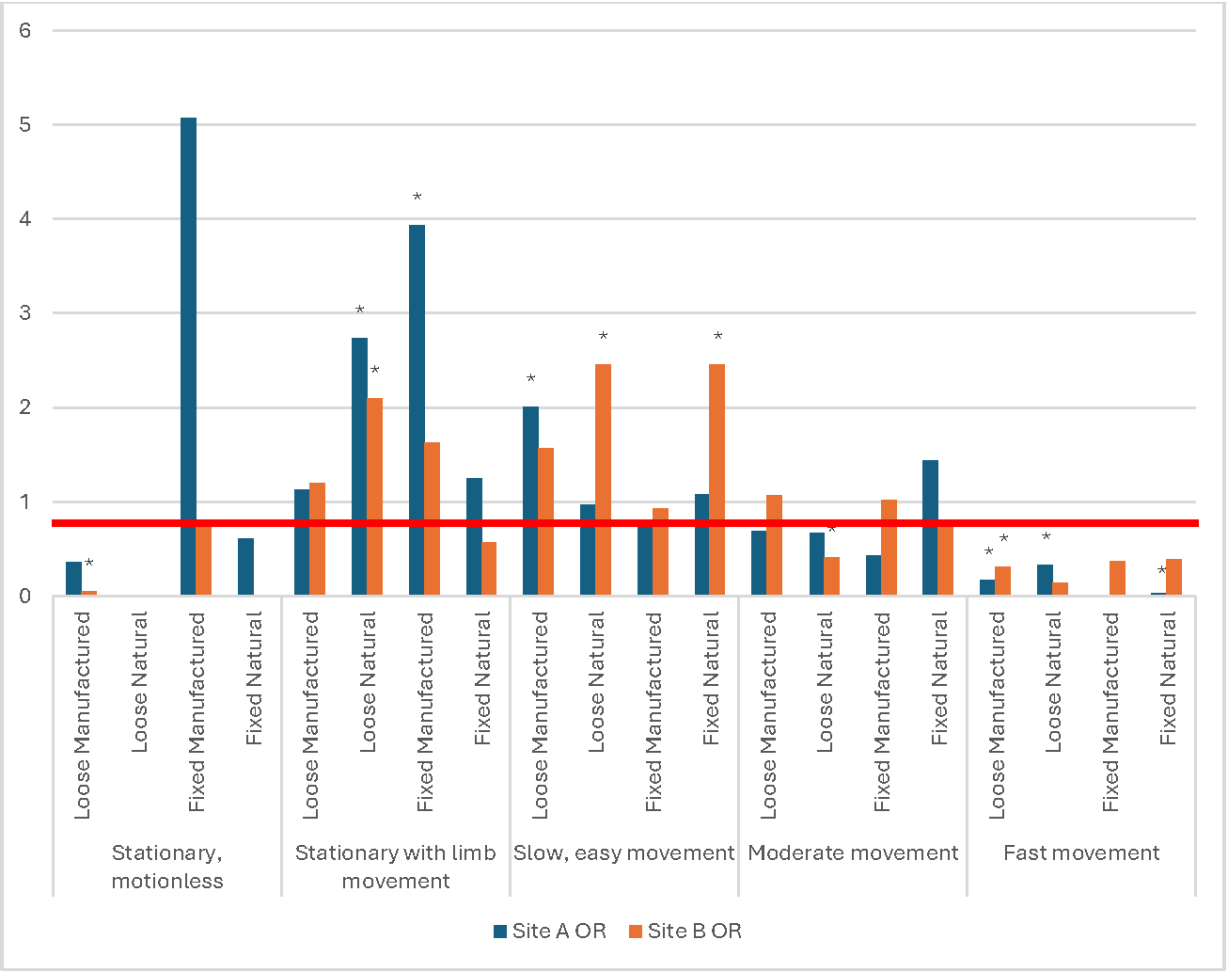
Odds ratios of loose parts and fixed feature use by physical activity intensity. Note: The y-axis displays odds ratios (unitless). Bars marked with an asterisk (*) indicate statistical significance (p < 0.05). Bars above the red line (OR > 1) represent increased odds, bars below the red line (OR < 1) indicate reduced odds, and bars at the red line (OR = 1) represent no association.

Table 3 and Figure 8 depict the odds ratios for the likelihood of specific types of loose parts and fixed features (manufactured and natural) being associated with different PA intensities (stationary-motionless, stationary-limb movement, slow easy movement, moderate movement, and fast movement) in play observations captured post-intervention (T6). Overall, play involving loose parts was more often associated with lower-intensity activity, particularly stationary activity with limb movement and slow/easy movement, compared with fast movement. This pattern suggests that loose-part play frequently involves manipulation, building, carrying, and re-arranging materials, behaviors that can be movement-rich without necessarily being high-intensity. Across sites, fast movement was generally less likely during play events involving loose parts and some feature types. Fixed features showed more mixed associations across intensity categories but tended to support a broader range of intensities depending on site context, with natural features showing stronger links to slower/easy movement in some cases. While both sites showed reduced likelihood of fast movement when loose parts were in use, Site B also showed clearer reductions in motionless sedentary behaviors when children engaged with loose manufactured materials.

**Table 3.** Associations of loose parts and fixed feature use with physical activity intensity.

	Site A				Site B		
Physical Activity Intensity	Type of Loose Part or Fixed Feature	Odds Ratio	Lower Bound	Upper Bound	Odds Ratio	Lower Bound	Upper Bound
Stationary, motionless	Loose Manufactured	0.36	0.08	1.61	0.05*	0.01	0.46
	Loose Natural	0.00	0.00	0.00	0.00	0.00	0.00
	Fixed Manufactured	5.07	0.86	29.74	0.79	0.08	7.56
	Fixed Natural	0.61	0.11	3.44	0.00	0.00	0.00
Stationary with limb movement	Loose Manufactured	1.13	0.72	1.78	1.20	0.67	2.15
	Loose Natural	2.74*	1.62	4.63	2.10*	1.06	4.14
	Fixed Manufactured	3.94*	1.74	8.91	1.63	0.86	3.10
	Fixed Natural	1.25	0.76	2.04	0.57	0.13	2.51
Slow, easy movement	Loose Manufactured	2.01*	1.41	2.88	1.57	0.95	2.59
	Loose Natural	0.97	0.61	1.54	2.46*	1.36	4.47
	Fixed Manufactured	0.76	0.34	1.71	0.93	0.51	1.69
	Fixed Natural	1.08	0.74	1.59	2.46*	1.02	5.93
Moderate movement	Loose Manufactured	0.69	0.47	1.02	1.07	0.70	1.66
	Loose Natural	0.67	0.40	1.15	0.41*	0.22	0.78
	Fixed Manufactured	0.43	0.14	1.28	1.02	0.61	1.71
	Fixed Natural	1.44	0.96	2.16	0.77	0.32	1.84
Fast movement	Loose Manufactured	0.17*	0.08	0.37	0.31*	0.15	0.63
	Loose Natural	0.33*	0.13	0.82	0.14	0.02	1.07
	Fixed Manufactured	0.00	0.00	0.00	0.37	0.11	1.26
	Fixed Natural	0.03*	0.00	0.22	0.39	0.05	3.08

Note: An odds ratio (OR) of 1 indicates no association. An OR greater than 1 suggests a positive association and higher odds, while an OR less than 1 indicates a negative association with lower odds. * p < 0.05

## 4. Discussion

Our purpose of this study was to observe and describe how an OLPP intervention was associated with children's FMS and PA, using a case study of two before- and after-school programs in Nova Scotia, Canada. Observational data were collected through behavioral mapping (BM) before the introduction of loose parts into outdoor play spaces (T0) and after integration (weeks 5–6; T6). In this discussion, will summarize findings within and between sites across the two timepoints. We will highlight key similarities and differences in children's FMS, PA, and engagement with loose parts and fixed features observed between the two sites, offering possible explanations for these variations, such as differences in outdoor environments and available affordances. Additionally, the strengths and limitations of the study will be discussed, followed by a consideration of the broader implications of these findings.

We used a case study approach for data collection and analysis. While the results will not be directly compared, contrasts between the two sites will be highlighted. Two distinct sites were chosen for this research. Site A was a suburban childcare center featuring a spacious outdoor area with several trees, grassy patches, shrubs, bushes, and a sandpit. The center's programming emphasized outdoor play and effectively integrated loose parts into its play space. In contrast, Site B was in an urban setting and had a very different play environment. Its outdoor area consisted of a large, hard-surface basketball court, a wooden platform with benches, and a small green space with just one tree and three garden boxes. This center offered more structured programming as part of its partnership with a community-based organization. Before the intervention, children's exposure to loose parts was limited to sports equipment like basketballs and soccer balls. At both sites, a significantly greater proportion of play events involved boys and children in Grades 1–3; at Site A, more play events involved older children (children in Grades 4–6), while younger children (Pre-Primary to Primary) engaged in a higher proportion of play events at Site B, specifically after loose parts were introduced.

### 4.1. Outdoor loose parts play at Sites A and B

The introduction of the OLPP intervention yielded different patterns of engagement across the two sites, resulting in varied observations of PA and FMS. At Site A, there was a significant decrease in the use of loose natural materials, while engagement with fixed natural features increased; the use of loose manufactured materials and engagement with fixed manufactured features did not change significantly. In contrast, at Site B, a higher proportion of observations involved loose manufactured materials (and loose parts use in general), while the use of loose natural materials, and engagement with fixed natural and manufactured features, did not change. Taken together, these patterns in loose parts use and feature engagement were associated with observed differences in children's FMS and PA intensity across sites and timepoints.

#### 4.1.1. Site A: Shift to stability-based play with fixed natural features

At Site A, overall, PA intensity remained stable following the introduction of loose parts, despite the introduction of diverse materials for children to play with. Observations during T6 favored stability-based movements rather than high-intensity locomotor movement or object control skills. These results were unexpected, as studies on open-ended materials often reported improvements in object control skills following a portable equipment intervention [40]. However, our observations align with those of Storli and Hagen, who found no change in PA behaviors in their study [41]. They examined children's day-to-day play and how their activity levels were influenced by the affordances offered in different outdoor play spaces [41]. They suggest that the affordances within the environment were fully utilized, which may explain the lack of change in PA levels. This rationale seems applicable to Site A, as the play environment incorporated loose parts before our study. Therefore, the variety of materials introduced may not have been sufficient to observe a measurable change in PA intensities or an increase in object control skills. Additionally, if the materials did not align with children's developmental needs or motor abilities, they may not have provided the appropriate level of challenge to encourage skill development. According to the affordance theory, skill acquisition depends on an environment that offers suitable opportunities for action (Gibson, 1979). If the materials lacked challenge or did not match children's abilities and interests, this could explain why there was no observed significant increase in object control skills. Thus, the intervention may not have provided enough opportunities for exploration and interaction, which are essential for developing FMS.

Conversely, a statistically significant increase in the observation of stability skills was noted. Stability skills, such as climbing, balancing, or squatting, were three times more likely to be used with fixed natural features, particularly trees. This association suggests that stability-based activities were prominently observed around fixed natural features like trees. This finding aligns with a study by Lim and colleagues, who audited 99 primary schools across five British Columbia districts to identify natural elements available to children and the FMS they afforded. The study revealed that forested areas, with features like trees, bushes, and stumps, are particularly conducive to stability skills, offering opportunities for balancing and climbing [42]. These natural elements appear to play a key role in encouraging stability-focused play, much like the fixed natural features at Site A. Our findings also indicate a consistent pattern of engagement with these fixed natural features over time following the intervention, potentially fostering greater confidence and competence in these skills, which contribute to the child's physical literacy development.

These findings could also be interpreted through the lens of the biophilia hypothesis, which suggests that children are naturally drawn to nature and its elements [43],[44]. Trees, in particular, offer an environment rich with sensory experiences and opportunities for physical challenge. Additionally, trees can provide a restorative effect, offering a more relaxing and calming atmosphere for children, which may encourage more sustained engagement [45]. Moreover, the attraction to natural elements like trees may be partly driven by children's innate desire for risk-taking in their play. Natural features such as trees present a range of physical challenges, such as climbing or balancing, which can be appealing to children seeking adventure and pushing their physical boundaries [45],[46]. These elements offer not only physical engagement but also opportunities for children to test their limits and capabilities.

It is also important to consider that baseline observations were collected in May, whereas post-intervention observations occurred in June and July. Warmer temperatures later in the season may independently influence where and how children play (e.g., increasing preference for shaded areas under tree canopies), which could contribute to greater engagement with trees and a corresponding shift away from sustained running-type activity toward lower-intensity, stability-oriented movement (e.g., balancing/climbing). As such, the increased tree engagement and stability-focused play observed at T6 may reflect a combination of intervention-related changes and seasonal/contextual conditions rather than the intervention alone.

We observed that stationary behavior with some limb movement was almost four times more likely to occur in conjunction with fixed manufactured features such as benches, suggesting that this association aligns with patterns typically observed during periods of rest. Stationary behavior with limb movement was also 2.74 times more likely to be observed with loose natural materials, supporting the idea that children engage in slower, more focused play when interacting with natural elements [41]. This type of play pattern, often involving mud, leaves, or sticks, suggests that children were engaging with these materials in a seated, exploratory manner. Interestingly, slow, easy movements were twice as likely to occur alongside loose manufactured materials, suggesting that even within a nature-based space like Site A, children's play with loose manufactured materials can adopt a slower, more concentrated pace. These findings suggest that natural and manufactured loose parts afford play that is less physically intense yet highly exploratory, with natural settings providing an environmental context for focused, slower-paced engagement.

#### 4.1.2. Site B: Shift toward more object-control, active play with loose manufactured items

In contrast, Site B exhibited a higher uptake of loose manufactured materials, which coincided with the significant increase observed in object control skills and moderate PA. The prevalence of object control skills aligns with findings from other studies associating portable equipment or loose materials in play settings with an increase in manipulation and object control movements [47]–[50]. Given that Site B had limited exposure to loose parts before the intervention, this increased engagement may be associated with a novelty effect [20]. Additionally, the outdoor play space at Site B, which had fewer fixed natural features compared to Site A, presented environmental affordances that favored loose manufactured materials that were strongly linked to object control skills and moderate PA. These observed patterns also align with the presence of a prominent asphalt play space, especially given the overall size of the outdoor area compared to Site A, where much of children's play occurred during the pre-intervention phase. This observation is further supported by our finding that object control skills were 5.5 times more likely to occur alongside loose manufactured materials at Site B. This aligns with the fact that most of the materials provided in the loose parts kits were manufactured items. Moreover, Site B's urban environment, with limited access to natural elements, may have established a setting where engagement with manufactured materials was more probable. Interestingly, loose natural elements, such as mud, were also observed being used in conjunction with fixed manufactured features like the mud kitchen. This integration suggests that children were finding creative ways to combine natural and manufactured materials, which reflects the versatility of these materials in facilitating different types of play.

It is also worth noting that Site B included a basketball court, which may have served as an additional space for higher-intensity, object control play. Observations suggest that children extended their use of loose parts into this area, further supporting the potential association between specific environmental features and observed FMS use. However, further research is needed to better understand how particular features of the environment may enhance the development of these skills.

Furthermore, stability skills, such as squatting, kneeling, or standing, were 1.28 times more likely to be observed with loose natural materials, particularly in the context of using mud, which seemed to encourage this type of engagement. Similar to Site A, we found that stability skills were 2.84 times more likely to be used with fixed natural features, such as trees. This finding is consistent with the biophilia hypothesis, which suggests that children are instinctively drawn to natural features [43]. The presence of natural elements like trees provides an environment that encourages physical exploration, balance, and risk-taking, which may explain the increased use of stability skills in these areas.

This leads toward more object control movement and more active play involving loose manufactured materials, suggesting that the materials provided coincided with a higher intensity of PA. These findings align with previous research, which suggests that certain equipment, such as hula hoops, can afford more intense PA compared to other materials [51]. Another study also revealed that portable equipment tends to increase moderate to vigorous physical activity levels [52]. What is particularly interesting is that this shift occurred in a more urban environment, which included a large basketball court. Studies have also shown that spaces with hard surface areas, like basketball courts, tend to encourage more running and dynamic play [53],[54]. This suggests that the built environment at Site B, including the availability of a large open manufactured space, may have provided the necessary context for the observed increase in moderate-intensity PA. The combination of the materials and physical space aligns with the observed variations in PA behaviors.

Additionally, we observed that stationary behaviors with limb movement was 2.10 times more likely to occur when children were interacting with loose natural materials; this pattern is characteristic of focused, controlled play while seated or stationary. This finding is consistent with the idea that natural materials, such as mud, leaves, or sticks, tend to promote slower, more deliberate forms of play [41]. Similarly, slow, easy movement was 2.46 times more frequently documented with loose and fixed natural elements, such as dirt and trees. This suggests that when children engaged with loose parts and natural features, they exhibited similarly slow, focused movements. Trees, as fixed natural features, may provide a grounding effect that encourages children to engage in low-intensity, exploratory play, potentially incorporating nearby loose materials, such as using sticks to build or create. Ultimately, the combination of loose materials and the physical space at Site B was associated with diverse play experiences, including a higher frequency of object control skills and higher-intensity play.

These contrasting patterns across sites suggest that the type of movements and PA intensity observed alongside loose parts are closely related to the pre-existing play affordances at a given location. At Site A, where natural elements already afforded stability movements, additional loose parts were not associated with a shift in PA intensity but rather aligned with continued stability-based play. At Site B, the outdoor play space was previously structured around sports equipment, and the introduction of new loose manufactured materials coincided with higher observations of object control skills and moderate intensity PA.

## 5. Strengths and limitations

### 5.1. Strengths

To our knowledge, we are the first to use the MOP observational measure [36] to understand FMS in the context of outdoor play and OLPP. While researchers have explored the broader impacts of outdoor play and OLPP on gross and fine motor skills [20],[25],[55],[56], our findings deepen the understanding by identifying the types of movements (locomotor, object control, stability skills) children use during these play activities. By focusing on the nuanced aspects of FMS, this research enhances our understanding of how outdoor play and OLPP facilitate various movement skills, thus addressing a crucial gap in the literature.

Similarly, research exploring how children's engagement with various types of loose parts is associated with PA behaviors and FMS is limited. While researchers have qualitatively observed the movements children exhibit during OLPP based on educators' perspectives [15],[16] and more broadly explored PA levels during OLPP (25), these findings are often descriptive, requiring additional quantitative evidence exploring how OLPP contributes to children's FMS and PA. Our study provides empirical evidence linking specific types of FMS and PA intensities to different loose parts; for example, the higher probability of object control skills occurring with loose manufactured items. By establishing these associations, we contribute valuable insights to the field, highlighting the capacity of loose parts to afford targeted movement skills and varying levels of physical engagement among children.

Our multi-site case study approach offers a comprehensive understanding of how an OLPP intervention was received across after-school centers, each characterized by distinct practices and outdoor play environments. For instance, one center had already embraced OLPP prior to the intervention, while the other site had a more structured approach to outdoor play, with little experience with and use of loose parts. This variability enables an in-depth exploration of how an OLPP intervention may differ based on existing practices and play space features. By highlighting these contrasts, our study highlights the adaptability of loose parts, demonstrating their potential to enhance play experiences in diverse outdoor environments.

### 5.2. Limitations

A key limitation to consider is that the way children perceive and interact with loose parts can vary greatly from day to day, influenced by factors such as mood, energy levels, social context, individual play needs, or environmental conditions. This variability means that our findings from this study, while insightful, may not be fully generalizable across all settings or populations. Children's engagement with affordances is not only shaped by the physical properties of the materials but also by individual factors such as developmental stage, prior experiences, and current emotional or social states [28],[57]. As a result, while the study offers valuable insights into how the use of different loose parts, and engagement with varied fixed features, can influence PA and FMS, the dynamic nature of affordances makes it difficult to draw broad, definitive conclusions about their impact across all children and contexts.

External influences such as adult involvement and access to loose parts can also shape children's engagement. As the broader PLEY School study collected data on adult interactions, researchers could further explore the role of adult facilitation or supervision in children's engagement with loose parts and the resulting PA and FMS outcomes. Moreover, environmental conditions such as weather may have influenced children's play behaviors; for instance, children observed later in the data collection period (T6), when it was warmer and tree canopies more developed, may have been more inclined to engage with natural features. While efforts were made to control for weather-related variation (i.e., observing play only during sunny/cloudy days, and not in the rain), it remains an important contextual factor to consider in future studies.

Another limitation of this study is its limited analysis of the age and gender of the play behaviors observed. Other research, such as that by Flannigan and colleagues, has indicated that age differences can significantly influence how children interact with loose parts [9]. For instance, Flannigan and colleagues found that when children engage with loose parts, older children were more likely to engage in role creation and take on leadership or decision-making roles, while younger children often adopted complementary or subordinate roles, responding to directions or tasks set by their older peers [9]. Interestingly, loose parts did not inherently restrict their use by age; instead, the open and unstructured nature of the materials supported inclusive play among mixed-age groups. These dynamics suggest that age may influence not just how children use loose parts, but also the types of social interactions and movements that emerge during this type of play. While we did collect data on children's perceived age group (based on school grade level) and gender as interpreted by trained observers during each observation, these demographic variables were not analyzed in relation to the outcomes of interest. Moreover, although informed written consent was obtained for all participating children, parent-reported age and gender of participating children were collected via a demographic questionnaire that was distributed to parents following the collection of the consent forms (following ethical protocol). While our team made every effort to collect demographic surveys of participating children, the timing of our study (tail end of the COVID-19 pandemic) created challenges in establishing contact with parents/guardians and follow-up, resulting in incomplete survey returns and missing parent-reported age and gender data. Future analyses could focus on exploring associations of PA and FMS with perceived age and gender (observer-reported), and may consider collecting non-sensitive demographics (e.g., age and gender) directly on the consent form while reserving more sensitive items for a separate survey.

Our study faced limitations regarding the number of participants, particularly at Site B, and to the structure of the BM data. Because BM data were collected using repeated observational scans rather than by tracking individual children over time, it is possible that the same child appeared in multiple scans if they remained in the observers' field of view across successive observation periods. If the same child was captured in multiple scans, the observations may not be fully independent, which can make the findings look stronger or more reliable than they actually are (e.g., smaller standard errors and narrower confidence intervals). Odds ratios should therefore be interpreted as observation-level associations rather than child-level effects. These considerations are most relevant at Site B, where the number of participating children was modest at baseline and smaller at follow-up, which may increase sensitivity to the play patterns of a small number of children and the risk of model instability and overfitting.

In contrast, other BM studies have included larger sample sizes. Coe and colleagues, for example, recruited 56 participants for a pre-post analysis of a renovated outdoor play space [47], while Mahfuzhoh and Marcillia observed 475 children in a single outdoor play setting [58]. Even studies with smaller participant pools, such as Cosco and colleagues who observed 30 children, established clear participant numbers [59]. Compared to these studies, our sample at Site B was limited. A larger sample size could provide greater insight into the variability in how children interact with loose parts, as well as the different PA and FMS that emerge from these interactions. For instance, children of different ages, genders, or cultural backgrounds may engage with loose parts in distinct ways, leading to varying opportunities for developing specific movement skills or participating in different intensities of PA. Including a broader range of participants would therefore improve the external validity of our findings by enabling us to better understand how the intervention may function across populations.

We focused on documenting the different types of movements observed during the intervention and did not assess the proficiency of FMS. As a result, we cannot determine whether the intervention led to improvements in the FMS of the children. What we can conclude is how children's engagement with loose parts in diverse outdoor environments contributed to their PA and FMS. Additionally, we were unable to capture the sequence of FMS performed during each play event (e.g. running followed by jumping and walking), or the sequence of use of loose parts (e.g. loose manufactured followed by loose natural), which limits our understanding of the way in which children combined FMS with loose parts use. Future research could benefit from video recordings to analyze these sequences in greater detail, providing deeper insights into the dynamics of movement patterns and the progression of FMS during OLPP.

## 6. Implications

Our findings of this study demonstrate how OLPP in suburban and urban play spaces support children's PA and FMS by providing diverse affordances and play opportunities unique to each setting. In a larger site with access to more natural features (e.g. trees, bushes) and prior engagement with OLPP, providing children with access to additional loose parts did not significantly alter children's PA but it did lead to a change in FMS expressed during play events, specifically more stability skills being used around the fixed natural features. Conversely, in a small site with more manufactured spaces (basketball court, parking lot) and less green space, and little prior use of loose parts, play events shifted from stationary and slow, easy play to more moderate intensity play involving object control skills with loose manufactured items, and significantly more loose parts play in general. While many findings were unique to each site, some similarities emerged. For example, children were significantly less likely to use locomotor skills when using loose natural and manufactured items, and significantly more likely to use object control skills when using loose natural and manufactured items and stability skills when engaging with fixed natural and manufactured features. Children were also more likely to engage in stationary activity with some limb movement when engaging with loose natural items, and less likely to engage in fast movement with loose manufactured items.

By highlighting how OLPP can contribute to children's PA and FMS in outdoor play environments with varying affordances for play, we can encourage stakeholders such as early childhood educators, before-and-after school program staff, school administrators, and policy-makers to include loose parts in other early years environments, creating richer play experiences that can support physical growth in children. Finally, this study enabled us to identify types of FMS (locomotor, object control, stability skills) and PA intensities associated with different types of loose parts and fixed features. These insights can inform targeted interventions and programming designed to enhance FMS and PA in children. For example, if a childcare center aims to shift time spent sedentary or in low-intensity PA to higher-intensity PA, and support children's object control skills, having loose manufactured materials like balls could be beneficial. If the aim is to provide slower, more immersive play experiences and support children's stability skills, it might consider providing opportunities for children to engage with loose natural and fixed natural elements (e.g. sticks, mud, and trees). However, early years stakeholders are encouraged to consider the fact that the richest play affordances and experiences, and greatest contributions to children's FMS and PA, will arise from the provision of diverse loose parts and outdoor play environments.

Although we focused on observed FMS and PA intensity, the findings can be interpreted within a physical literacy lens that emphasizes not only physical competence, but also the affective elements that support lifelong engagement (e.g., motivation and confidence) [16],[60]. The open-ended and child-directed nature of loose parts may support the motivational and confidence-related dimensions of physical literacy by encouraging choice, experimentation, and desired challenges within familiar play spaces. While motivation and confidence were not directly measured in this study, our results suggest that designing after-school environments with diverse fixed features (e.g., trees for stability-rich play) and adaptable loose materials may create conditions that support not only skill practice and activity, but also the positive movement experiences that can underpin sustained PA participation over time.

Our findings also align with provincial priorities in Nova Scotia. The Let's Get Moving Nova Scotia action plan explicitly identifies a goal to “help early childhood educators (ECE) enhance their skills and knowledge about physical activity, outdoor play, and physical literacy,” alongside actions that support affordable before- and after-school programs and encourage the use of indoor and outdoor environments to increase movement [61]. Our results provide applied evidence that OLPP may be a feasible way to advance these priorities in an after-school setting. Specifically, following OLPP implementation, children's play shifted in ways that reflected richer movement experiences. Taken together, these patterns suggest that OLPP may help educators and program staff move beyond simply having children play outside toward intentionally supporting physical literacy through child-led play, without requiring highly specialized equipment or highly structured programming. In resource-limited programs, OLPP may also be comparatively scalable because it can draw on durable, reusable, and community-sourced materials [62]. Educator support could be delivered through brief training focused on facilitation, risk–benefit approaches to outdoor play, and simple strategies for rotating and maintaining materials directly matching the Let's Get Moving plan's emphasis on strengthening educator knowledge and skills for outdoor play.

From an implementation perspective, our findings suggest that OLPP can be scaled in after-school programs and can be most effective when paired with educator support that builds confidence in facilitating child-led outdoor play. This aligns with the Loose Parts Play Toolkit [62], which aims to increase children's access to loose parts and explicitly emphasizes increasing adults' confidence and providing practical guidance for introducing loose parts play across a range of settings, including out-of-school care such as after-school programs. In practice, after-school programs could adopt a starter kit and facilitation approach where they select a small set of loose parts suited to the site (e.g., more loose manufactured materials in smaller manufactured spaces to support object-control opportunities; and pairing loose natural materials with fixed natural features to support stability-rich play), and deliver a brief staff orientation focused on how to facilitate this type of play. This approach provides an implementation that complements the context-dependent patterns observed in this study.

In future studies, researchers could use BM through a person-centered lens to explore how children engage with loose parts and how their FMS evolve through this type of play. While tracking individual development over time presents some challenges, such as isolating the impact of loose parts from natural, age-related developmental changes, this approach could offer valuable insights into how children's movement patterns diversify and progress in environments with loose parts. This type of research could offer a description of the types of movements that emerge and evolve through repeated engagement with loose parts, contributing to a deeper understanding of how this type of play supports FMS development.

While we used a case study approach to provide rich, detailed descriptions of two distinct sites, expanding this work to include a broader range of settings would offer valuable insights into loose parts play. Moreover, examining how the integration of loose parts in diverse environments is associated with children's PA and FMS could help identify context-specific strategies to optimize outdoor play experiences for children.

Researchers should also consider how individual characteristics such as age, gender, and ethnicity may influence children's engagement with loose parts, and how these factors interact with PA and FMS outcomes. These individual characteristics may influence how children experience and benefit from OLPP. Building on this, researchers could also focus on understanding how environmental features support OLPP. This could involve exploring specific features, documenting the types of loose parts used, and examining the associated FMS and PA intensities to better understand how outdoor play spaces can be optimized to support children's development.

## 7. Conclusions

In this study, we observed and described the association between an OLPP intervention and children's PA and FMS across two after-school programs in Nova Scotia, Canada, with distinct outdoor play spaces. The findings highlight that loose parts do not universally influence the intensity of children's PA or FMS; rather, observed patterns align with existing play affordances and the types of materials and features provided by the outdoor play space. At Site A, PA intensity remained stable, while a higher proportion of stability skills and a lower proportion of object control skills were documented. These results suggest that the outdoor play space already contained several natural features and loose parts, which provided affordances for play. Children were observed spending time engaging with fixed natural elements (e.g. trees and bushes) and with loose natural materials (e.g. sticks and mud). In contrast, Site B showed a significant increase in moderate PA, alongside higher frequencies of object control skills and lower frequencies of locomotor and stability skills. The introduction of manufactured loose parts (e.g., basketballs, tires, and pool noodles) coincided with more frequent object manipulation and moderate-intensity PA. These findings highlight the context-dependent nature of OLPP interventions and emphasize that not all loose parts equally contribute to PA and FMS engagement. Future OLPP research should entail the role of environmental features, children's prior exposure to loose parts, and seasonal influences on children's PA and FMS. By providing insights into how loose parts relate to PA and FMS, educators, program coordinators, and policymakers can make more intentional choices about the materials they provide or incorporate within the outdoor play space, thereby maximizing the benefits of outdoor play for children's physical and motor skill development.

## Use of AI tools declaration

The authors declare they have not used Artificial Intelligence (AI) tools in the creation of this article.


