# Supplementary material for: Observing children's outdoor loose parts play, fundamental movement skills, and physical activity in after-school programs through a behavioral mapping approach
Source: AIMS Public Health. 2026 Apr 20;13(2):513–45. doi: 10.3934/publichealth.2026027 (PMC13368684; doi:10.3934/publichealth.2026027)
Supplement: Supplementary file 1 [file publichealth-13-02-027-s001.pdf]

AIMS Public Health

http://www.aimspress.com/journal/aimsph

AIMS Public Health, 13(2): 513–545.
DOI: 10.3934/publichealth.2026027
Received: 08 January 2026
Revised: 19 March 2026
Accepted: 27 March 2026
Published: 20 April 2026

Research article

Observing children's outdoor loose parts play, fundamental movement skills, and physical activity in after-school programs through a behavioral mapping approach

Nila Joshi[1,*], Son Truong[1], Janet Loebach[2], Daniel Rainham[1,3], Becky Feicht[1] and Michelle Stone[1]

[1] School of Health and Human Performance, Dalhousie University, Nova Scotia, Canada
[2] Cornell College of Human Ecology, Cornell University, New York, United States of America
[3] Centre for Planetary Health and Sustainable Care, Nova Scotia, Canada

* **Correspondence:** Email: Nila.Joshi@dal.ca; Tel: +9024941167.

Supplementary

Appendix A

This section describes the three categories of FMS: locomotor skills, object control skills, and stability skills. The following table provides a clear overview of these categories, including specific movements within each category. The definitions below have been adapted from other descriptors.

Locomotor Skills:

Locomotor skills are described as movements where the body moves through space from one point to another point and include movements such as walking, running, or jumping. The acquisition of locomotor skills exhibits a predictable developmental progression [70].

Object Control Skills:

Object control skills involve the body's ability to manipulate an object, moving it from one point to another [70]. Specifically, object control skills include a range of movements that require precision and accuracy in manipulating, catching, throwing, and striking objects. Proficiency in object control skills is important for individuals to engage in various activities such as writing, drawing, and participation in sports.

Stability Skills:

Stability skills are a set of motor abilities focused on maintaining balance, stability, and postural control during both static and dynamic movements such as balancing, stretching, or bending [70]. These skills are important in providing individuals the opportunity to participate in a wide range of activities, including sitting, standing, walking, running, jumping, and engaging in sports.

Movement Observations during Play (MOP)

**Category**	**Movement**	**Definition**
**LOCOMOTOR SKILLS**		
Crawl	Crawling	Starting in all-fours position, opposite limbs move in conjunction with one another in a smooth, consistent rhythm.
Dancing	Dancing	Dancing is an extension of spontaneous movement to music
	Expressive Movement	Movements that manifest themselves during various (particularly emotional) psychological states and that serve as their external expression
Run	Running	Running is an extension of walking, except there is a flight phase when neither foot is on the ground
Jump/Skip	Galloping	A forward step followed by a leap on the trailing foot. By definition, this pattern must be performed in the front-facing direction, and the same leg always leads
	Hopping	A form of jumping in which the propelling force is generated in one leg and the landing is accomplished on the same leg
	Skipping	The skip consists of a forward step followed by a hop on the same foot (an uneven rhythmical pattern)
	Jumping	Exploding off the ground with the legs in a forward and upward direction, landing on both feet
	Step up*	Stepping onto an elevated surface using one leg and then stepping back down
	Step down*	Stepping down from an elevated surface with one leg and returning to the starting position
Walking	Marching	Arms and leges move in contralateral pattern with a consistent tempo; to move along steadily, usually with a rhythmic stride and in step with others.
	Walking	To move along on foot, with the advancing foot moving opposite to swinging arm
Swim	Swimming or playing in a pool.	Moving in water; motor activities performed in water for any purpose
Rolling	Roll	Rolling forward: Hands and arms receive body weight evenly; the chin is tucked to the chest and the head slides through. The weight shifts from the arms to shoulders and the back curves. The body curls through and weight moves to the feet
Ride	Scooter	Footboard mounted upon two or more wheels controlled by an upright steering handle and propelled by the user in an upright position

	Skateboarding	A footboard mounted upon two or more wheels and is usually propelled by the user who sometimes stands, sits, kneels, or lies upon the device while it is in motion.
	Cycling	A vehicle with two wheels tandem, handlebars for steering, a saddle seat, and pedals by which it is propelled
	Roller Skating	A pair of shoes, mounted upon wheels, propelled by the user in an upright, crouched, or kneeling position.
Other	Sliding*	Sliding is an asymmetrical gait that consists of a step on one foot, then a leap-step on the other foot; movement is in sideways direction.
**MANIPULATIVE/OBJECT CONTROL**		
Sending and Receiving*	Kicking	Kicking involves imparting force to an object with the foot.
	Throwing	Throwing involves releasing an object forcefully with the hands
	Catching	The actions of bringing an airborne object under control by using the hands and arms
Pull/Push	Pulling an object	Exerting force onto an object to make it move toward the source of the force
	Pushing an object	To move an object away by pressure
Roll	Rolling ball or object*	Using an underarm action to project an object
Other	Strike*	Striking involves swinging at and hitting an object with a part of the body or an implement
	Dribbling with hands*	Bouncing the ball with one hand
	Dribbling with feet*	Maintaining control of the ball with feet
	Lifting*	Moving an object from its initial position upwards
	Holding*	To have or maintain something in the grasp; to support in a particular position or keep from falling or moving
	Carrying*	An object remains lifted and is moved horizontally without mechanical assistance
	Digging*	To break up, turn, or loosen (earth) with an implement
	Stirring*	To mix or disturb the relative position of the particles or parts of especially by a continued circular movement
	Shaking*	Shaking (object): to briskly move something to and from or up and down, especially in order to mix
**STABILITY SKILLS**		
Lie Down	Lying	Refers to being in a horizontal position on a supporting surface.
Sit/squat	Kneeling	A position where at least one knee is in contact with some part of the environment (usually the ground), and the body weight is being supported predominantly through the knees.
	Squatting	A position when the knees are fully flexed and the back of the thigh rests against the calf muscles while keeping the heels flat on the ground.
	Sitting	A position in which one's weight is supported by one's buttocks rather than one's feet, and in which one's back is upright.
Stand	Standing	A position in which one has or is maintaining an upright position

		while supposed by one's feet.
Rough and tumble	Rough and tumble play	A form of higher energy play where children limb over each other, wrestle, roll around and pretend to play fight in the spirit of fun.
Swing	Swinging on a swing	To move freely to and from especially in suspension from an overhead support
Climb	Climbing	Movement taking place in the direction opposite to gravitational forces in which humans actively use the upper part of the body.
	Hanging	To remain suspended or fastened to some point above without support from below
Rock	Rocking on a teeter totter or rocking horse	Two children or groups of children ride on opposite ends of a plank balanced in the middle so that one end goes up as the other goes down
Other	Balancing*	Static balance involves a stable centre of gravity that remains within the base of support (ex., standing in one place). Dynamic balance involves maintaining control and balance while moving (ex., walking on a beam).
	Shaking (body)*	A short, quick, vibrating movement in a body part or the whole body.

Note: Movements marked with a * indicate additions to the measure. Initially, there were 17 movements, which the author expanded upon based on the definitions provided by the OSRAC-E tool [71]. Fourteen more movements were added, and an additional 15 were included from the literature review, consultations with stakeholders, and reliability rounds.

AIMS Press

© 2026 the Author(s), licensee AIMS Press. This is an open access article distributed under the terms of the Creative Commons Attribution License (http://creativecommons.org/licenses/by/4.0)
